# Exploring the anti-biofilm effect of darobactin B and colistin in static and dynamic environments

**DOI:** 10.1128/spectrum.02868-25

**Published:** 2026-01-08

**Authors:** Flaviana C. Susanto, Zerlina G. Wuisan, Marius Spohn, Till F. Schäberle, Michael Marner

**Affiliations:** 1 Institute for Insect Biotechnology, JLU Giessen, Giessen, Germany; 2 Fraunhofer-Institute for Molecular Biology and Applied Ecology (IME) Branch for Bioresources, Giessen, Germany; 3 German Center for Infection Research (DZIF), Partner Site Giessen-Marburg-Langen, Giessen, Germany; Yan'an University, Yan'an, Shaanxi, China

**Keywords:** *Pseudomonas aeruginosa*, microfluidic, biofilm assay, anti-biofilm, multidose regime, darobactin B, colistin

## Abstract

The increasing challenges of treating biofilm-associated infections highlight the importance to develop new options against biofilm-embedded bacteria. In this study, we evaluated darobactin B—a promising pre-clinical antibiotic candidate targeting the outer membrane protein BamA—against *Pseudomonas aeruginosa* biofilms. First, a static biofilm assay was used for primary screening and showed that darobactin B reduced the viable cells within a biofilm by six orders of magnitude compared to the untreated control. Subsequently, a microfluidic model was conducted to allow real-time monitoring of biofilm development, treatment, and potential regrowth under controlled hydrodynamic conditions. In this assay, the reference compound colistin exhibited strong activity after single-dose administration. Although less effective after single dosing, darobactin B showed notable anti-biofilm activity by killing 85%–97% of biofilm cells and suppressing biofilm recovery for at least 48 h when applied repeatedly once daily for 5 days. When administered repeatedly in the multi-dose regimen, darobactin B caused biofilm disruption and cell detachment, leading to ~40% surface coverage reduction. Our findings highlight the potential of darobactin B as an anti-biofilm agent.

**IMPORTANCE:**

Bacterial biofilms pose serious healthcare challenges, contributing to chronic and device-related infections. Biofilm-embedded bacteria are highly resistant to conventional antibiotics and lead to growing reliance on last-resort drugs, thus underscoring the need for new therapeutic approaches. This study shows that darobactin B consistently disrupts *Pseudomonas aeruginosa* biofilms and delays regrowth. The multi-dose microfluidic assay provides a flexible platform for real-time evaluation of biofilms, which may support the optimization of treatment regimens.

## INTRODUCTION

Biofilms are microbial communities encased in a self-produced matrix of extracellular polymeric substances. This matrix facilitates cell-surface attachment and enables the bacteria to thrive in extreme environments (1), increasing the resistance of biofilm-embedded cells to antibiotics by 10–1,000 times compared to their free-floating forms (2). Moreover, metabolic inactivity (3) and phenotypic changes (4), as a consequence of limited nutrient and oxygen availability in deeper regions of biofilms, further impair the activity of many antimicrobial agents. Approximately 80% of chronic and nosocomial bacterial infections are related to biofilms (5). One of the most studied bacterial species in this context is *Pseudomonas aeruginosa* (6, 7). This pathogen is a major cause of infection in cystic fibrosis (CF) patients, where biofilm formation leads to airway obstruction and ultimately, respiratory failure in the diseased lung (8, 9). In addition, biofilm formation on medical devices, such as tracheal intubation tubes, plays a major role in ventilator-associated pneumonia (VAP) (10). The prevalence of antimicrobial resistance among *P. aeruginosa* isolated from CF and VAP patients is particularly high, even for the latest approved drugs (11, 12).

Although some antibiotics have been shown to reduce the biomass of *P. aeruginosa* biofilms (13), colistin (CST) is one of the few agents with reported activity against less metabolically active cells in deeper regions of biofilms (14, 15). However, CST is classified as a last-resort antibiotic due to its nephrotoxicity and neurotoxicity under suboptimal dosing (16). An increasing number of reports on CST-resistant strains (17–19) further highlight the urgent need to identify new treatment options for biofilm-related infections, preferably with a new mode of action (20).

One promising candidate could be darobactin B (DAR B) (21), a recently discovered antibiotic that selectively kills Gram-negative bacteria. By binding to BamA, the central component of the β-barrel assembly machinery (BAM) protein complex at the outer membrane, DAR B restricts maturation and insertion of outer membrane proteins, leading to dysfunctionality of the membrane (22, 23). While DAR B has shown promising activity against several clinically isolated *P. aeruginosa* strains (11), it has not been comprehensively tested against biofilms. To address this gap, we evaluated the anti-biofilm potential of DAR B using static and dynamic *in vitro* biofilm assays.

First, we used a microplate-based biofilm eradication assay to determine the primary activity of the test compounds against *P. aeruginosa* ATCC 27853. In addition to the surface coverage, the biofilm viability was quantified using LIVE/DEAD fluorescence staining and correlated with colony-forming unit (CFU) counts for validation. Static models are widely used due to their simplicity and scalability (8, 24, 25), but they are limited to single endpoint measurements and do not consider shear stress, which influences the volume, thickness, surface area, and overall stability of the biofilms (26–28). To overcome these limitations, we implemented microfluidic-based dynamic biofilm models, allowing continuous nutrient flow and waste removal, as well as real-time monitoring of biofilm development and antibiotic response (5). By precisely controlling the flow rate (and thereby hydrodynamic shear stress), microfluidic systems can create physical conditions that closely mimic the natural environment of pathogens (29). Recently proposed methods, like the BioFlux system (30), BiofilmChip (31), and other custom-built platforms (32), have a similar workflow with three main phases: cell attachment, biofilm formation, and compound treatment (31, 32). We included an additional regrowth phase in our setup to capture resistant subpopulations (33, 34) in deeper layers that may reform the biofilm after antibiotic treatment.

Single-dose experiments were conducted to assess immediate anti-biofilm activity, before we performed a multi-dose regimen, in which the established biofilms were treated with five doses of DAR B over 5 days, followed by the regrowth analysis. The workflow might serve as a transitional step from a static plate-based assay to an *in vivo* setting. Ultimately, the study highlights the potential of DAR B as a promising antibiotic candidate with anti-biofilm activity.

## MATERIALS AND METHODS

### Bacterial strain and growth conditions

Working cryo-cultures of *P. aeruginosa* ATCC 27853 were preserved on lysogeny broth (LB) agar pieces in 50% glycerol at –80°C. Cells were activated in LB (Carl Roth, Karlsruhe, Germany) and incubated overnight (37°C, 180 rpm, 85% relative humidity [RH]) before being used in subsequent microplate or microfluidic assays.

### Preparation of antibiotic solutions

DAR B was provided by the Natural Product Research Group of the Justus Liebig University Giessen, Germany. Stock solutions of DAR B and Penicillin G (PEN, Biomol, Hamburg, Germany) were prepared in sterile ultrapure water, whereas CST (TOKU-E, Bellingham, WA, USA) was dissolved in dimethyl sulfoxide (DMSO, Carl Roth).

For the static microplate assays, serial dilutions of CST (256–0.5 μg/mL) and DAR B (1,024–2 μg/mL) were prepared in LB. In the microfluidic experiments, each antibiotic solution was adjusted to a concentration of 8× the minimum inhibitory concentration (MIC), with the exception of PEN (due to inactivity against the test strain). The final concentrations were 64 µg/mL PEN, 2 µg/mL CST, and 64 µg/mL DAR B.

### Antimicrobial susceptibility testing

The activity of the antibiotics against *P. aeruginosa* ATCC 27853 was evaluated using broth microdilution assays in agreement with Clinical and Laboratory Standards Institute (CLSI, Pittsburgh, PA, USA) guidelines with only minor modifications as previously described (11). In cases where the MIC varied between replicates, the higher MICs were used for the biofilm experiments. Differences between replicates never exceeded more than one dilution step. Overnight cultures (37°C, 180 rpm, 85% RH) in LB were adjusted to the McFarland 1 standard (corresponds to 3 × 10^8^ CFU/mL, approximate OD_600_ = 0.17–0.24) and diluted to an inoculum density of 5 × 10^5^ CFU/mL. Each dilution series of the antibiotics (64–0.03 μg/mL) was tested in triplicate. Wells containing cell suspensions without test compounds were used as growth controls. The background reading was obtained by averaging five replicates of the medium-only control per test plate. The assay plates were incubated for 18 h (37°C, 180 rpm, 85% RH), and the growth of the test strain was quantified by measuring the turbidity of each well at 600 nm using a LUMIstar Omega microplate spectrophotometer (BMG Labtech, Ortenberg, Germany). The MIC was defined as the lowest concentration inhibiting cell growth by at least 85% relative to the growth control.

### Static microplate-based biofilm eradication assay

The activity of antibiotics against *P. aeruginosa* ATCC 27853 biofilms was initially evaluated in static 96-well microplates. The overnight culture was adjusted to an OD_600_ of 0.04. A volume of 100 µL was added to black clear-bottom microplates (Greiner, Frickenhausen, Germany). The assay plate was incubated for 18–24 h (37°C, without agitation, 85% RH) to allow biofilm formation.

After incubation, the inoculum was gently aspirated to remove the spent media and planktonic cells, leaving only the adhered biofilm. Each well was then gently rinsed with 100 µL sterile phosphate-buffered saline (PBS). Next, a dilution series of test samples (100 µL/well) was added with a multichannel pipette at an angle of ~45° toward the well wall to avoid contact with the biofilm (24). Due to the inactivity of PEN against the test strain, only dilution series of CST and DAR B were tested in quadruplicate. Wells containing untreated biofilms served as growth controls. Medium background was averaged from 10 wells per plate. After 6 h incubation (RT, without agitation, 85% RH), the well content was aspirated to remove the antibiotic mixture and cells detached as a consequence of the treatment.

Biofilm viability was assessed using fluorescence staining with 2.5× SYBR Green I (Invitrogen, Thermo Fisher Scientific, Waltham, MA, USA) and 5 µM propidium iodide (PI, Carl Roth) in sterile PBS. A 100 µL of the staining mixture was added to each well at an angle of ~45° toward the well wall and the plate was incubated in the dark for 1 h (RT, without agitation, 85% RH). Finally, 90 µL of the well content was aspirated, leaving ~10 µL/well. Fluorescence images of each well were captured using Cytation 5 Cell Imaging Multimode Reader (Biotek, Agilent Technologies, Santa Clara, CA, USA) equipped with a 1.25× objective at a resolution of 1,496 × 1,496 pixels and 32-bit depth. Green fluorescent protein and Texas Red filters were used to visualize viable (green) and membrane-compromised (red) biofilms, respectively.

To validate this approach and to avoid false positives from PI binding to extracellular DNA (eDNA) that would mimic cell death (35), we investigated biofilm viability by subsequent CFU determination. Colony counts were determined in triplicate of the wells treated with the six highest antibiotic concentrations, representing a range from fully eradicated to unaffected biofilms (CST 256–8 µg/mL and DAR B 1,024–32 µg/mL), as well as untreated and media controls. To do so, 90 µL of sterile LB was added to each well and mixed until biofilms were homogeneously resuspended after the fluorescence readout. Subsequently, 10-fold dilution series were prepared, and a 10 µL aliquot of the *P. aeruginosa* suspensions was plated on LB agar. After incubation for 18 h at 37°C, colonies were counted and expressed as log_10_ CFU/mL. Only plates containing 30–300 colonies are considered countable. Plates with >300 colonies are not quantified. If multiple dilutions were within the countable range, the dilution with a count closest to 100 was used.

### Microfluidic model and experimental setup

**Fig 1:**
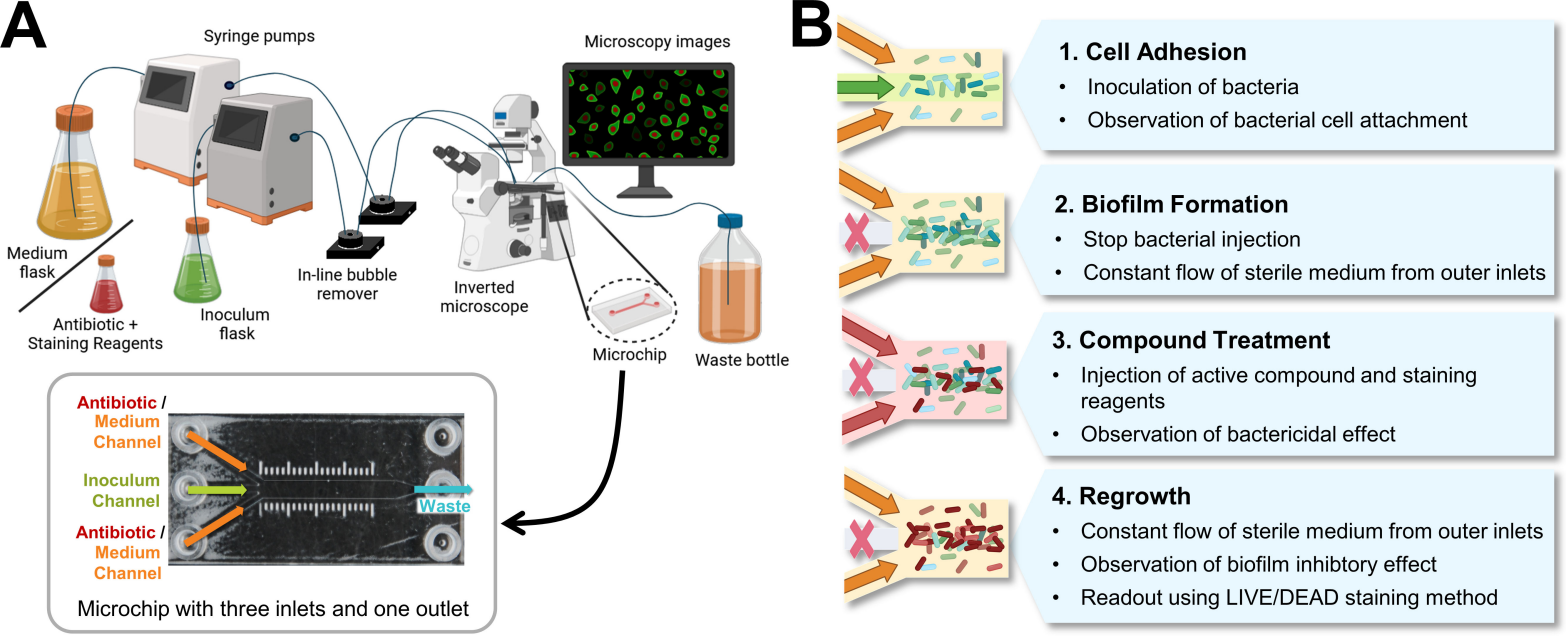
(**A**) Experimental setup of the microfluidic assay, including the layout of the microfluidic chip. The chip consists of three inlets and one outlet. Using syringe pumps, growth medium and antibiotic reagents are pumped through the outer channels, while the bacterial inoculum is introduced through the middle inlet. The microchip is placed on an inverted fluorescence microscope to enable real-time monitoring of the experiment. (**B**) Four major steps in the microfluidic experimental workflow: cell adhesion, biofilm formation, compound treatment, and regrowth (modified from reference 32). For the multiple-dosage experiment with DAR B, the compound treatment step was repeated five times with a 24-h interval. Figures were created using BioRender (37, 38).

The setup is based on a commercially available ibiTreat 3-in-1 µ-Slide chip (ibidi, Gräfelfing, Germany) with three inlet channels that converge into a single observation chamber and one outlet channel. This design restricts intensive cell adhesion and biofilm growth on the sidewalls of the chamber, where the flow rates and shear stress are lower (32, 36). Growth medium, bacterial inoculum, and antibiotic solutions are supplied to the chip via syringe pumps. The chip was placed on top of an inverted fluorescence microscope to visualize the biofilm. Like in the static assay, SYBR Green I/PI cell staining was used to investigate the viability of the biofilm cells. The experimental setup is shown in Fig. 1A.

The ibiTreat 3-in-1 µ-Slide chip features a plasma-treated (hydrophilic) surface, which facilitates cell adhesion (https://ibidi.com/content/207-chamber-surfaces). Each chip consists of three 1,000 µm wide and 400 µm high rectangular inlet channels that converge into a 23.73 mm long, 3,000 µm wide, and 400 µm high observation chamber, followed by an outlet channel identical in dimensions to the inlets. The microchip has a total channel volume of 60 µL (https://ibidi.com/channel-slides/55--slide-iii-3in1.html).

LB medium and bacterial suspension were independently pumped into the chip using two different microfluidic Mitos-Duo-XS syringe pumps (Dolomite Microfluidics, Royston, UK). Sterilized in-line air bubble traps (ELVEFLOW, Paris, France) were installed to prevent bubbles from entering the microfluidic chip. The syringe pumps, bubble traps, and microfluidic chip were connected via 1.6-mm polytetrafluoroethylene tubing (Darwin Microfluidics, Paris, France). Before and after each experiment, the entire system was sterilized with 10% hydrogen peroxide (Carl Roth), sterile ultrapure water (0.055 μS/cm), and 70% isopropanol. All experiments were carried out at room temperature. To capture images at each phase of the experiment, the chip was placed on a Zeiss Axiovert 200M inverted fluorescence microscope equipped with a 40×/1.3 oil objective, fluorescence and bright-field illumination, and a digital microscope camera (SPOT Imaging, Sterling Heights, MI, USA).

### Biofilm formation of *P. aeruginosa* ATCC 27853 in microfluidic system

To prepare the bacterial inoculum for the microfluidic assay, overnight *P. aeruginosa* ATCC 27853 cultures were filtered through a sterile 40 µm Nylon cell strainer (Corning Incorporated, Corning, NY, USA) to avoid cell clumps and diluted to OD_600_ = 0.2 in LB before injection into the microfluidic system.

This experiment was conducted to establish suitable growth conditions within the microfluidic system, focusing on the first two phases of the general workflow: cell adhesion and biofilm formation (Fig. 1B). First, LB was flushed through the two outer inlets to prime the tubing and chamber. Then the bacterial suspension was introduced via the middle inlet at 100 µL/min for 90 min, while sterile LB was pumped through the outer channels at 350 µL/min. The higher flow rate of the outer channels confined the bacterial attachment to the center of the chip, which is defined as our study area. It also removes non-adherent cells, ensuring that only the attached cells are present in the observation area (32). Following the cell adhesion phase, the inoculum flow was stopped, and the experiment proceeded with the biofilm formation phase. The flow rate of the outer channels was increased to 400 µL/min to observe biofilm formation under hydrodynamic conditions for 48 h. Images of bacterial adhesion and biofilm formation were recorded at 0, 8 to 24, 36, and 48 h from 10 different locations along the study area using a bright-field filter.

### Single-dose antibiotic treatment

The experimental workflow comprises four major phases: bacterial cell adhesion, biofilm formation, antibiotic treatment, and regrowth (Fig. 1B). The bacterial cell adhesion and biofilm formation phases were carried out as described above.

After 16 to 18 h of biofilm growth, sterile LIVE/DEAD staining solution without antibiotics was applied at 200 µL/min for 30 min to ensure the biofilm viability. Next, we administered the antimicrobial test compounds at a dose of 8× MIC mixed with the same staining solution at a moderate flow rate of 200 µL/min for 3 h.

Images from five to ten different locations along the study area were recorded every 10 min with red and green fluorescence filters to detect PI (maximum excitation at 535 nm and maximum emission at 617 nm) and SYBR Green I (maximum excitation at 497 nm and maximum emission at 520 nm) signals, respectively.

SYBR Green I stains all cells by binding to DNA, while PI can only enter cells with a damaged membrane. If cell membranes are disrupted, both dyes can bind to the DNA, and PI may partly replace SYBR Green I due to its stronger affinity for nucleic acid binding (39). Nevertheless, under our flow condition and dye ratio, PI could not completely replace SYBR Green I. Initially, all cells were stained green. If a treatment led to membrane disruption, cells gradually showed additional red staining, leading to an orange-to-red color in the overlay images. Hence, the ratio of the membrane-compromised (red) fluorescence signals and total cells was calculated based on the area occupied by the cells in the captured fluorescence images as a proxy for biofilm viability.

After antibiotic treatment, the flow was stopped. The antibiotic reservoir was replaced with sterile LB, which was supplied through both outer channels at 400 µL/min for 48 h to allow regrowth. Biofilm regrowth was monitored at 0, 8, 24, and 48 h post-antibiotic administration by injecting the staining solution. Data were processed and analyzed as described below (section “Image pocessing”).

### Multiple doses of darobactin B treatment

In this experiment, the treatment phase was modified, while the other phases remained identical to the single-dose experiments. During the treatment phase, the biofilm on the chip was subject to five times compound administrations with 24-h intervals. Each treatment began with the introduction of a staining solution without antibiotics for 30 min to evaluate the biofilm viability. Subsequently, DAR B (64 µg/mL mixed in the same solution) was applied for 2 h at 200 µL/min. In the period between treatments, LB was supplied through the two outer channels at 400 µL/min. During each 2-h treatment, images from five different locations along the study area were taken every 10 min with red and green fluorescence filters. The antibiotic treatments were then followed by the regrowth phase as detailed above.

### Image processing

In the microplate-based assays, fluorescence images were analyzed using BioTek Gen5 software (BioTek, Agilent Technologies, Santa Clara, CA, USA), in which the area of viable (green), membrane-compromised (red), and total cells was automatically quantified and reported as physical area in square microns (µm²).

For the microfluidic assays, fluorescence and bright-field images were processed using ImageJ v1.54f (40). Biofilm surface coverage and the ratio of red-to-total cells were calculated over time and visualized as scatter plots using the GraphPad Prism v8.0 (GraphPad Software, Boston, MA, USA).

### Statistical analysis

For the microplate-based assays, data are presented as mean ± standard deviation (SD) from at least three independent replicates unless stated otherwise. Statistical comparisons of CFU counts between treatments and untreated controls were performed using one-way ANOVA, followed by Dunnett’s multiple comparison test in GraphPad Prism v8.0 to compare each treatment group with the control. Differences were considered statistically significant at *P* < 0.05.

## RESULTS

### Observation of CST and DAR B activity against *P. aeruginosa* biofilm in static assay

**Fig 2:**
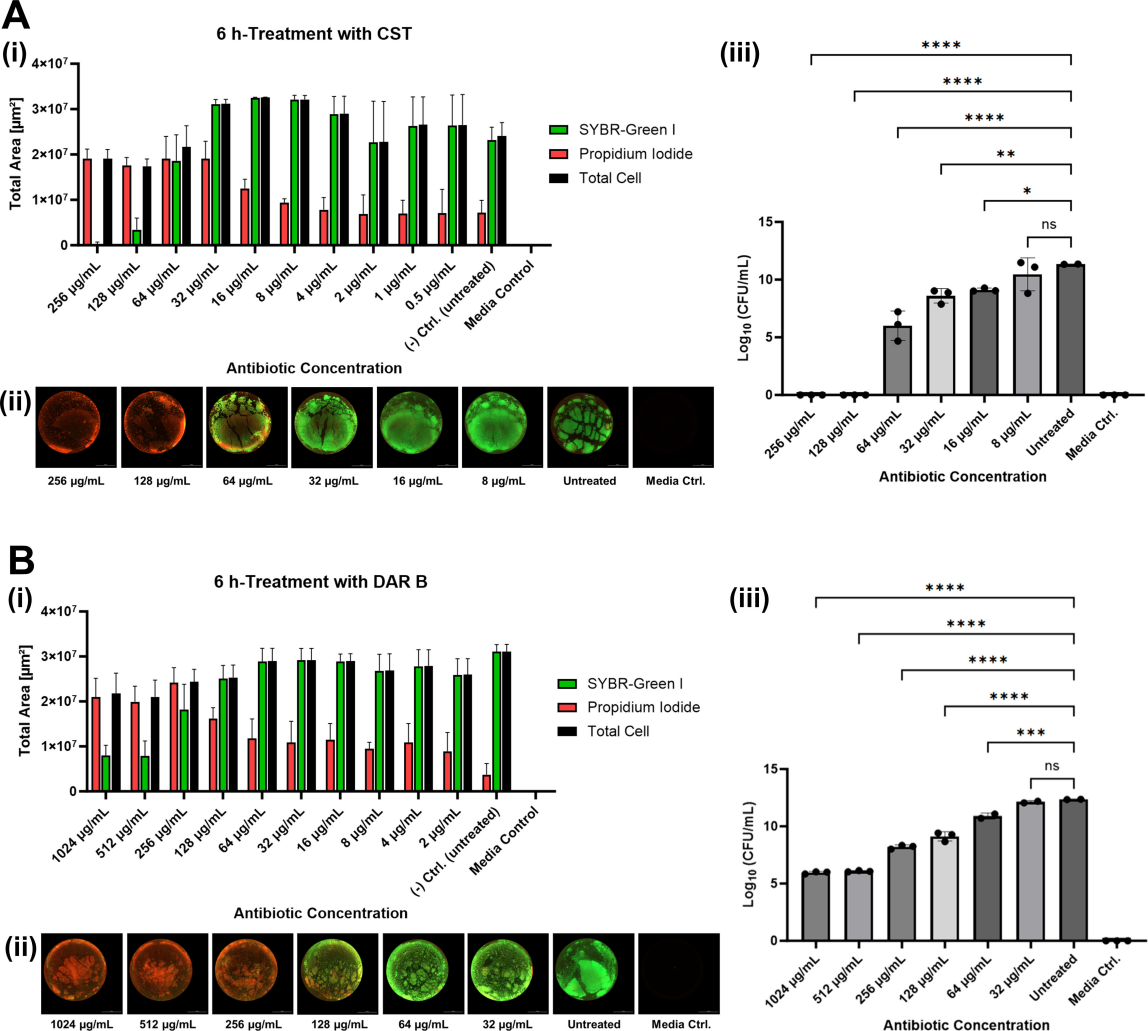
Results of microplate-based static assay. *P. aeruginosa* ATCC 27853 biofilms after 6-h treatment with colistin (**A**) and darobactin B (**B**): bar graphs showing the mean ± standard deviation of calculated area of red-stained, green-stained, and total cells of the biofilms after the treatment (i); representative fluorescence images (ii) (images of full assay plates are available in Fig. S2); and CFU count results (iii). Statistical analysis shows significant differences between the treatments with the five-highest antibiotic concentrations compared to untreated biofilms (****P < 0.0001, ***P < 0.001, **P < 0.01, *P < 0.05; ns, not significant).

The anti-biofilm activities of CST and DAR B toward *P. aeruginosa* ATCC 27853 biofilms were first evaluated in static microplate-based biofilm eradication assays, followed by CFU quantification. CST visibly reduced the total cell area from 3.2 × 10^7^ µm^2^ at 8 µg/mL to 1.7–1.9 × 10^7^ µm^2^ at 256 and 128 µg/mL, indicating partial detachment of the biofilms (Fig. 2A[i]). Moreover, >99% of the total cell area was stained red at the two highest concentrations, implying that almost all cells exhibited damaged membranes. In comparison, DAR B required higher doses to achieve a similar effect (Fig. 2B[i]). From the total cell area, DAR B treatment at 1,024–512 µg/mL resulted in >94% red-stained cells. However, the remaining green-stained area was slightly greater compared to CST-treated biofilms (7.8–8.0 × 10^6^ µm^2^ at ≥512 µg/mL DAR B versus 0.3–3.40 × 10^6^ µm^2^ at ≥128 µg/mL CST). This indicates moderate, yet significant activity of DAR B on the biofilm.

At lower concentrations, both antibiotics showed decreased anti-biofilm activity as represented by the decreasing ratio of red-fluorescent to total cells. The area of red-stained cells in biofilms treated with ≤8 µg/mL CST or ≤32 µg/mL DAR B was not significantly different than the area measured in the untreated control. Untreated biofilms showed high biofilm viability, with >96% of green-stained (>2.30 × 10^7^ µm^2^) and <30% red-stained area (<7.20 × 10^6^ µm^2^). These observations were consistent with the representative fluorescence images (Fig. 2A[ii] and B[ii]). No fluorescence was detected in the medium control, thereby eliminating the possibility of dye autofluorescence.

During subsequent CFU determination, no colonies were recovered from biofilms treated with ≥128 µg/mL CST, corresponding to an 11-log_10_ reduction compared to the untreated biofilms (Fig. 2A[iii]). In contrast, the highest concentrations of DAR B (1,024 and 512 µg/mL) did not eradicate all biofilm cells, but reduced CFU counts to ~1 × 10^6^ CFU/mL, representing a 6-log_10_ decrease compared to the untreated biofilms (Fig. 2B[iii]). Both CST and DAR B showed a clear concentration-dependent anti-biofilm effect: for CST, a strong increase in CFU/mL was observed between 128 and 64 µg/mL (approximately 6-log_10_), followed by a 1- to 3-log_10_ increase for each subsequent twofold dilution. Similarly, observed CFU/mL increased 1- to 2-log_10_ per DAR B dilution step. Statistical analysis showed a significant difference between the untreated control and treatment with the five highest tested concentrations of CST (256–16 µg/mL) and DAR B (1,024–64 µg/mL), resulting in *P* < 0.05 and *P* ≤ 0.001, respectively. In summary, CST achieved complete eradication at the concentration of 128 µg/mL, while small subpopulations persisted in DAR B-treated biofilms. Nevertheless, DAR B showed significant activity at ≥64 µg/mL, confirming its potential as an anti-biofilm agent.

### Examination of *P. aeruginosa* biofilm development in the hydrodynamic condition

**Fig 3:**
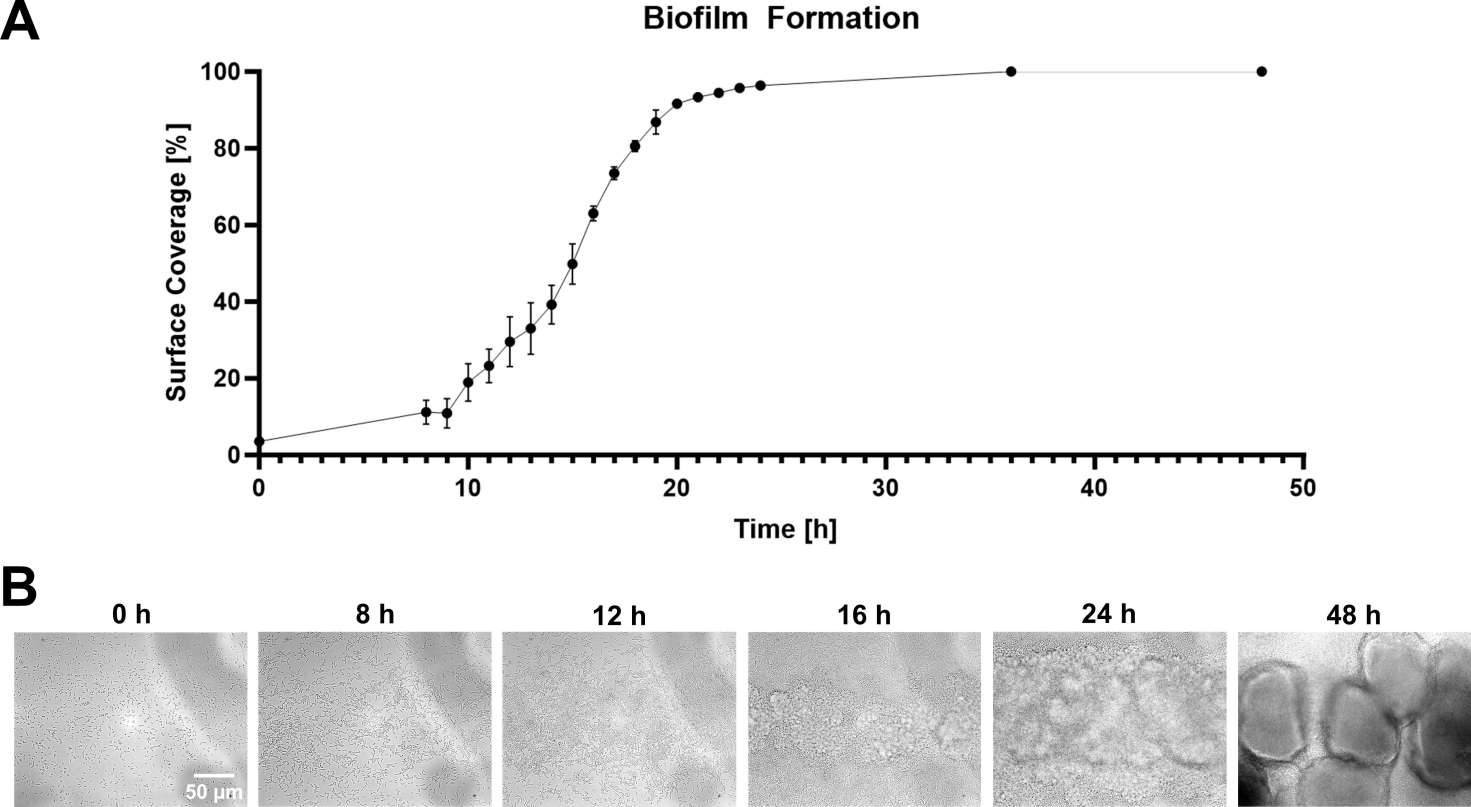
Analysis of *P. aeruginosa* biofilm formation. (**A**) Surface coverage of *P. aeruginosa* ATCC 27853 during the biofilm formation step. (**B**) Representative bright-field microscopic images of various developmental stages of *P. aeruginosa* ATCC 27853 biofilms: initial attachment (0 h), strong adhesion (8 h), early developmental stage (microcolony formation—12 h), maturation (macrocolony formation—16, 24, and 48 h).

Biofilm development of *P. aeruginosa* ATCC 27853 was monitored under controlled flow conditions in the microfluidic system (Fig. 1A and B). All stages of *P. aeruginosa* biofilm maturation can be observed within 48 h (Fig. S3A). Bacterial cell attachment was observed during the inoculation phase, followed by the cell division and the formation of a bacterial monolayer after 8 h. Surface coverage increased to 11% within this period, followed by an exponential increase in cell colonization, resulting in ~70% coverage after 16 h (Fig. 3A). The biofilm entered the maturation phase, forming multi-layer microcolonies after 12 h, and then macrocolonies after 18 h. After 24 h, >90% of the surface was covered by the biofilm and by 48 h, we observed a characteristic mushroom*-*like biofilm architecture (Fig. 3B; Fig. S3B). Bright-field microscopic images of several focal positions were taken, qualitatively showing the increasing thickness of the biofilms in the late developmental stages (Fig. S3C). Based on this timeline, we selected an incubation period of 16–18 h for biofilm formation in our workflow.

### Single-dose treatment of PEN, CST, and DAR B using microfluidic assay

Established biofilms were exposed to a single 3-h treatment with 8× MIC of CST, DAR B, and PEN, followed by a 48-h regrowth phase in antibiotic-free medium. PEN was used as a negative control and tested at a concentration of 64 µg/mL (Table S1 and Fig. S1).

**Fig 4:**
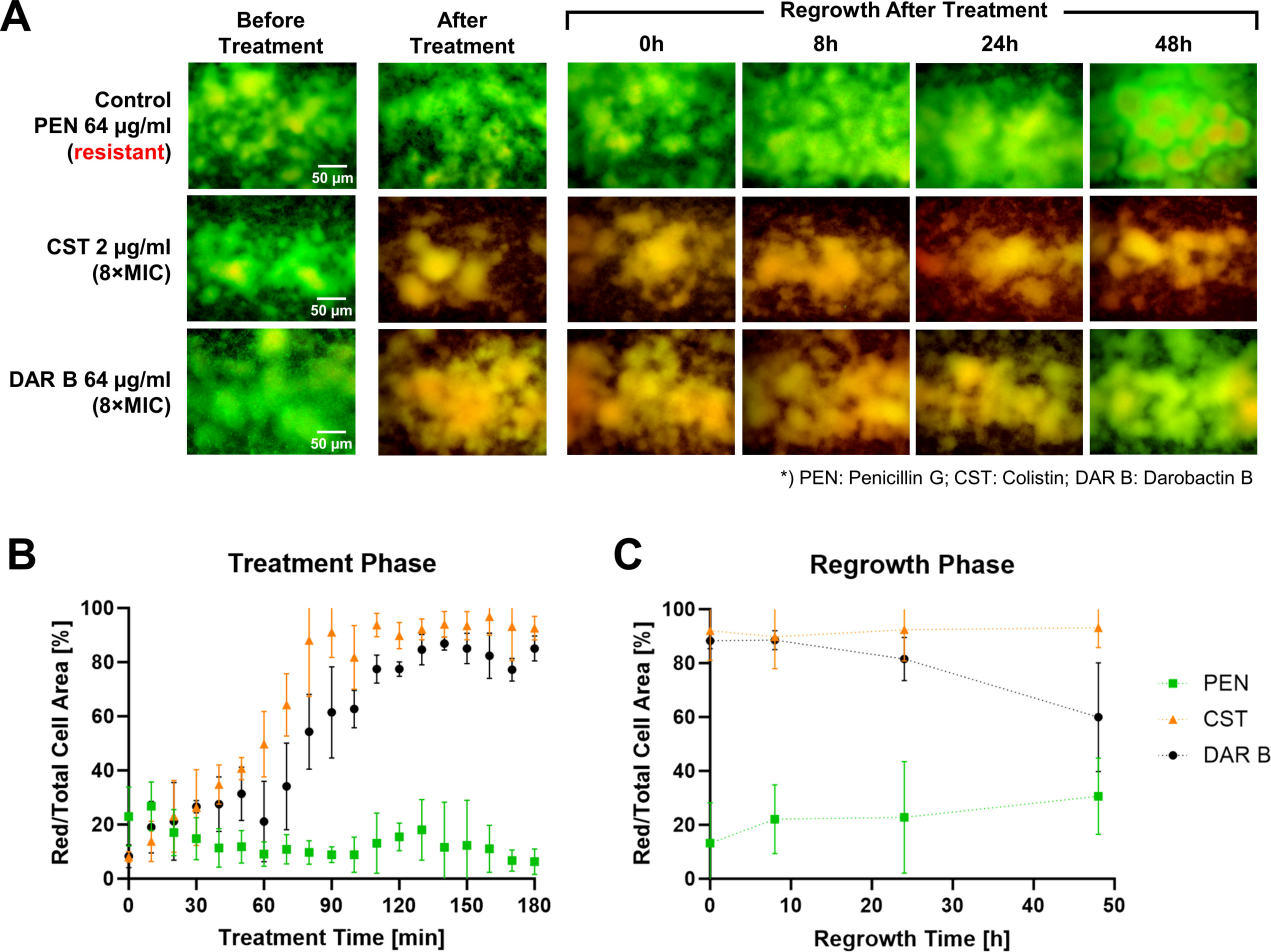
(**A**) Biofilms of *P. aeruginosa* ATCC 27853 stained with SYBR Green I and PI before and after antibiotic treatment, as well as during the regrowth phase. Bottom: effect of PEN, CST, and DAR B on mature *P. aeruginosa* ATCC 27853 biofilms during the 3-h treatment (**B**) and during the regrowth phase (**C**). The ratio of membrane-compromised (red-stained) cells to total cells was calculated over time during the treatment and regrowth phases.

As expected, only a small fraction of the biofilms exhibited orange-red coloring before the treatment started, indicating high viability of the biofilm population (Fig. 4A). PEN showed no activity, i.e.*,* the cells were unaffected and continued to proliferate until 48-h (Fig. 4A), with a constant low level of PI-stained cells (~6%–27%, Fig. 4B). Hence, the biofilm cells were neither killed nor detached over time due to the influence of inactive treatment, the constant flow, or the microfluidic chip environment itself.

In comparison, treatment with CST or DAR B killed most of the biofilm cells (Fig. 4A). We found that treatment with DAR B killed >80% of the cells within 120 min, whereas CST killed >90% within 90 min (Fig. 4B). Biofilms treated with DAR B showed a reduced proportion of the membrane-compromised cells of ~60% at 48 h post-treatment, indicating biofilm regrowth (Fig. 4C). In contrast, no regrowth occurred after treatment with CST, suggesting effective penetration of the biofilm matrix and elimination of the cells within. In line with the membrane-disruptive mode of action of CST, the ratio of membrane-compromised cells to total cells remained high (>90%) until the end of the experiment. These findings are consistent with the preliminary trials in which the biofilm treatment and regrowth were recorded only from one single location of the chip (see Fig. S4).

### Multiple-dosage treatment of DAR B using microfluidic assay

Next, we investigated the effect of sequential biofilm treatment with DAR B. The regimen was selected based on the single-dose studies, where >80% of the cells appeared dead after 120 min of DAR B exposure (Fig. 4B), with no recovery within the following 24 h (Fig. 4C). Thus, treatment duration of 120 min with an interval between doses of 24 h was selected.

**Fig 5:**
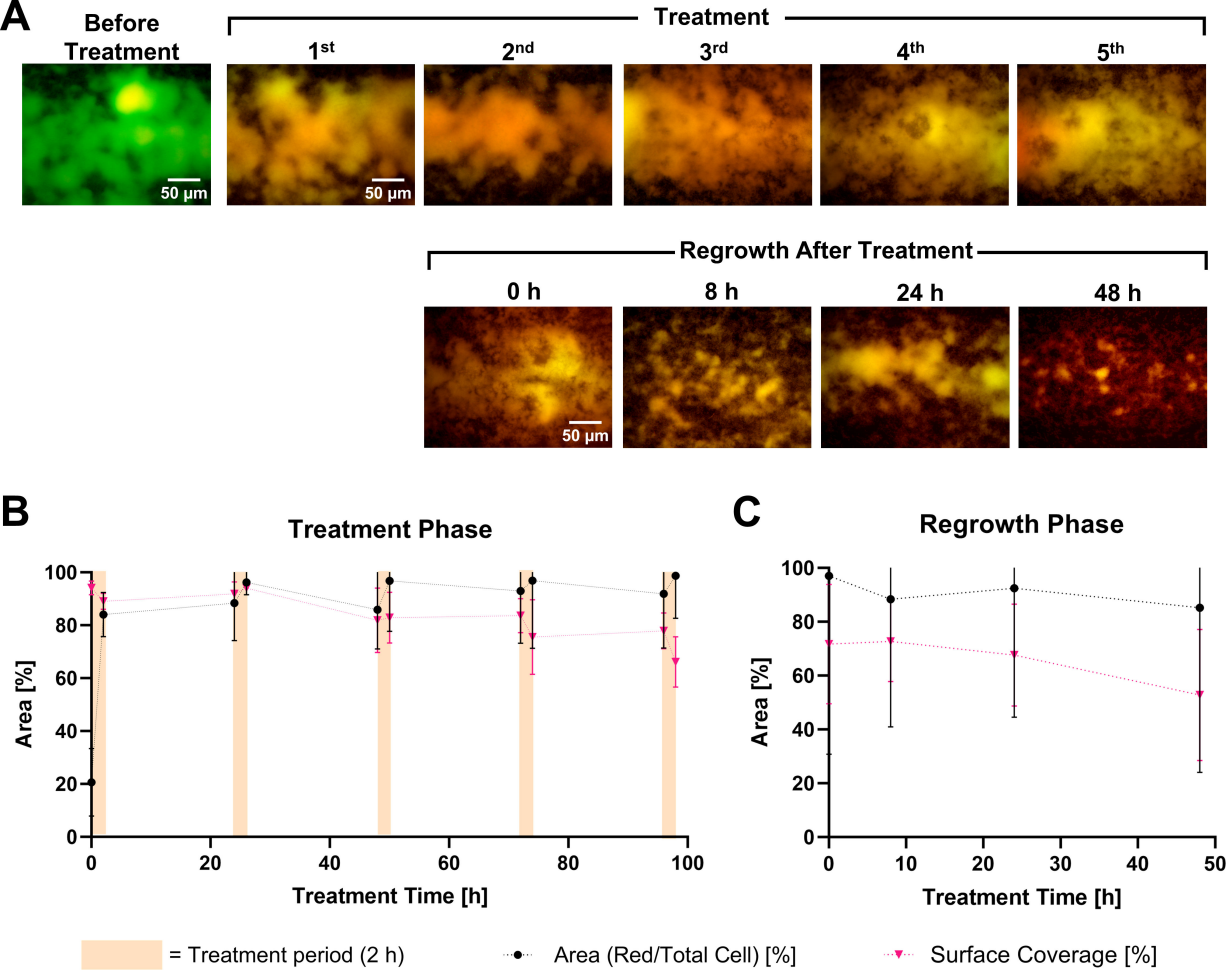
(**A**) Representative microscopic images of *P. aeruginosa* ATCC 27853 biofilms stained with SYBR Green I and PI before and after treatment(s) with 64 µg/mL DAR B, as well as during the 48-h regrowth phase in the multidose experiments. Each treatment involved the application of 64 µg/mL DARB for 2 h with a 24-h interval. Bottom: effect of DAR B on mature *P. aeruginosa* ATCC 27853 biofilms during the sequential 2-h treatments (**B**) and the regrowth phase (**C**). The ratio of membrane-compromised (red-stained) cells to total cells and percentage of surface coverage were calculated over time during the treatment and regrowth phases (simplified; detailed calculations of each 2-h treatment are shown in Fig. S6).

A preliminary feasibility study, in which images were captured from a single field in the chip, was performed using three doses of DAR B (co-administered with staining reagents) under the same treatment duration and interval. Although the biofilm partially recovered by 48 h post-treatment, this regimen resulted in ~60% reduction of surface coverage (Fig. S5). Intrigued by the technical feasibility and biological response, we continued to evaluate the potential of DAR B to disrupt the biofilms and delay regrowth by extending the regimen to five doses. Similar to the single-dose experiment, cell viability was quantified by image recording in various locations within the study area under fluorescent filters (Fig. 5A).

Before treatment, most of the cells were viable, as indicated by only a small fraction of red co-stained cells (21%) and >90% surface coverage. After the first dose of DAR B, 84% of the biofilm appeared orange-red, indicating extensive membrane perturbation in most cells (Fig. 5B). The proportion of membrane-compromised cells remained at >90% across subsequent doses, although minor short-term regrowth was detected between the first and second, as well as the second and third doses. At these intervals, the percentage of red-stained cells decreased to 88% and 86%, respectively. Biofilm surface coverage gradually declined after each treatment, reaching ~60% after the final dose (Fig. 5B).

No recovery was observed throughout the 48-h regrowth phase. The ratio of red/total cell area remained high (85%–97%), confirming sustained membrane damage and lack of biofilm regeneration. At the end of this phase, the biofilm surface coverage further decreased to 53%, representing >40% reduction compared to the initial coverage (Fig. 5C). The lack of viable cell recovery within this period indicates that DAR B successfully delayed and/or suppressed biofilm regrowth, although some structural remains of the biofilms persisted.

## DISCUSSION

Infectious diseases associated with biofilms pose a major threat in the healthcare sector, because biofilm-embedded cells are resistant to many traditional antibiotics. In this study, we set out to assess the anti-biofilm activity of antimicrobial agents under static and dynamic conditions. The primary microplate-based (static) assay allowed screening of multiple compounds and concentrations. Once the compound activity was identified, we examined DAR B and CST in a dynamic microfluidic model. Real-time analysis tracked *P. aeruginosa* ATCC 27853 biofilm development—from initial attachment to micro- and macrocolony maturation (41) and identified the optimal timing for treatment initiation. While our study focused on the killing and/or elimination of well-established biofilms, this technology could also be applied to target specific developmental stages of the biofilm life cycle. Comparative studies on gene essentiality showed that each stage is associated with different gene expression patterns, representing adaptations to the changing microenvironment within the biofilms (42, 43). For example, early stages require genes for initial adhesion (42) and motility (43, 44), whereas later maturation relies on genes for matrix production (42, 44) and stress adaptation (42, 43). Understanding these stage-specific functional requirements could help to identify weak points in biofilm development, potentially reveal new drug targets, and ultimately support the development of more targeted anti-biofilm strategies.

The different responses of mature *P. aeruginosa* biofilms to CST and DAR B illustrate the complexity of biofilm eradication. CST was used as a benchmark due to its well-known membrane-disruptive mechanism and anti-biofilm effect. Most antibiotics fail to eradicate persister cells in the inner-biofilm layer, because traditional molecular targets, including those involved in growth, protein synthesis, or cell wall biosynthesis, are non-essential during dormancy (45). In contrast, CST kills Gram-negative bacteria by physical membrane disruption: the polycationic peptide part of the antibiotic interacts with lipid A, while the N-terminal lipophilic side chain inserts into bacterial membranes, leading to increased permeabilization and ultimately leakage. This mechanism does not rely on metabolic activity and could thereby also affect dormant cells. Consistent with this, our study shows that CST effectively killed biofilm-encased, presumably metabolically inactive, *P. aeruginosa* cells. Although CST is widely used for treating multidrug-resistant *P. aeruginosa* infections in CF patients (46), other studies reported the development of resistant subpopulations within biofilms and ultimately regrowth (46–48). This highlights the importance of alternative treatment options for effective biofilm eradication in the future.

One promising candidate is DAR B, as it showed consistent anti-biofilm activity in both static and hydrodynamic models. Under flow conditions, a single dose appeared to kill most of the cells, although the biofilms subsequently recovered in the regrowth phase (Fig. 4C). With five sequential doses, DAR B strongly suppressed biofilm regrowth for at least 48 h and triggered extensive detachment, reducing surface coverage by ~40% (Fig. 5C). The sustained effect of DAR B observed in the multi-dose experiments points to a sequential, layer-by-layer clearance rather than an immediate eradication after a single dose as seen for CST. Different from physical membrane disruption, the novel mode of action of DAR B is based on the inhibition of the transmembrane protein BamA, a bacterial insertase that catalyzes the folding and insertion of outer membrane proteins (22). Accordingly, DAR B, like most other antibiotics, targets metabolically active cells in the upper biofilm layers, where BamA is essential. Killing surface cells might disrupt the biofilm matrix, expose deeper layers to nutrients, and resuscitate dormant cells to make them susceptible to subsequent doses, thus driving progressive biofilm removal. In this sense, a further refined multi-dose DAR B regimen may eradicate *P. aeruginosa* biofilms completely and warrants further evaluation. Increasing treatment number and duration or shorter intervals between doses may further improve the anti-biofilm activity. Moreover, potential synergies with quorum-sensing inhibitors, enzymes (49), and/or other antibiotics such as CST, as well as formulations based on lipid nanoparticles (50), could improve the biofilm activity.

While we found no DAR B-resistant colonies, a prior report showed that *Escherichia coli* can acquire darobactin resistance via point mutations in the gene coding for BamA that diminish drug-target interaction (22). Interestingly, the same study demonstrated that darobactin-resistant BamA mutants exhibit slower growth and reduced virulence *in vivo* (22). Although both CST and DAR B attack the outer membrane integrity of Gram-negatives, their target and mode of action differ substantially. Hence, DAR B might be effective against the CST-resistant strains and thereby represent a promising contribution to fight antimicrobial-resistant biofilms—in mono or combinatorial therapy.

It has to be noted that the usage of fluorescence staining as the sole method to determine microbial biofilm viability might be susceptible to artifacts, such as PI-eDNA staining. To prevent false conclusions by overestimating the share of membrane-compromised cells, additional endpoints, such as CFU determination or biofilm regrowth, should be included in anti-biofilm screening designs. Furthermore, the study focused on only one *P. aeruginosa* strain. While ATCC 27853 is a generally accepted reference strain for antimicrobial evaluations, future studies would benefit from including clinical isolates featuring different antibiotic resistance profiles and biofilm phenotypes. In this context, the experimental workflow could be used to investigate drug efficacy against such isolates in axenic or polymicrobial biofilms (31). Furthermore, the multiple dosage workflow could be used as a basis for designing antibiotic dosing regimens, including antibiotic dose levels, treatment durations, and dosing intervals. From an ethical perspective, this technology has the potential to reduce animal experimentation during preclinical development by facilitating the transfer of primary *in vitro* results to *in vivo* experiments.

## SUPPLEMENTAL MATERIAL

Supplemental material spectrum.02868-25-s0001.docx Table S1; Fig. S1 to S6.

ASM does not own the copyrights to Supplemental Material that may be linked to, or accessed through, an article. The authors have granted ASM a non-exclusive, world-wide license to publish the Supplemental Material files. Please contact the corresponding author directly for reuse.
