# Supplementary material for: Exploring the anti-biofilm effect of darobactin B and colistin in static and dynamic environments
Source: Microbiol Spectr. 2026 Jan 8;14(2):e02868-25. doi: 10.1128/spectrum.02868-25 (PMC12889053; doi:10.1128/spectrum.02868-25)
Supplement: Supplemental material — Table S1; Fig. S1 to S6. [file spectrum.02868-25-s0001.docx]

# **SUPPLEMENTARY**

Table S1 – MIC and selected antibiotic concentration of Penicillin G (PEN), colistin (CST), and darobactin B (DAR B) used in the microfluidic assay against P. aeruginosa ATCC 27853

| **Antibiotic** | **Minimum Inhibitory Concentration (MIC) [µg/mL]** | **Concentration used in microfluidic assay (8× MIC)**  **[µg/mL]** |
| --- | --- | --- |
| PEN | > 64 (resistant) | 64 |
| CST | 0.25 | 2 |
| DAR B | 8 - 4 | 64 |


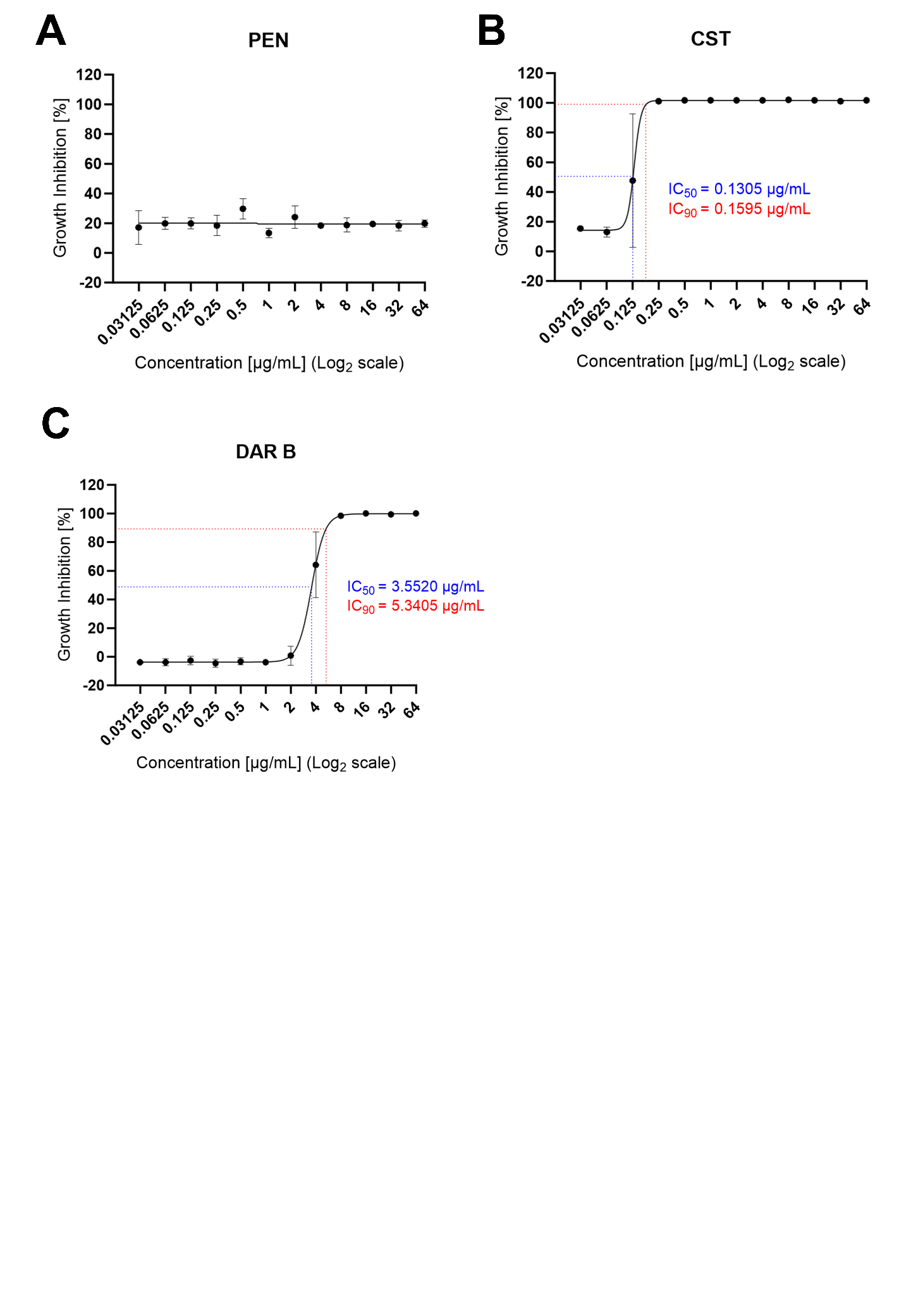


Figure S1 – Dose-response curve of the tested antibiotics including the calculation of relative IC_50_ and IC_90_


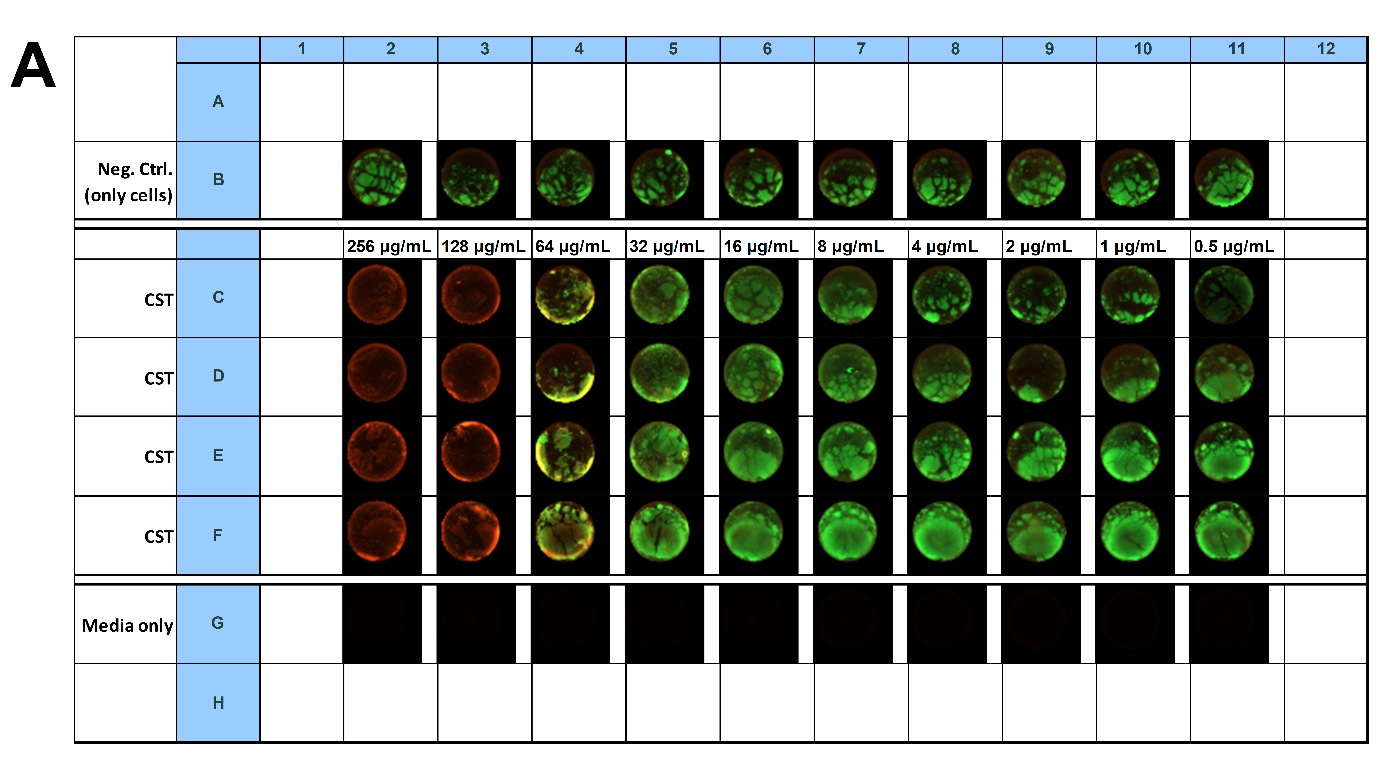


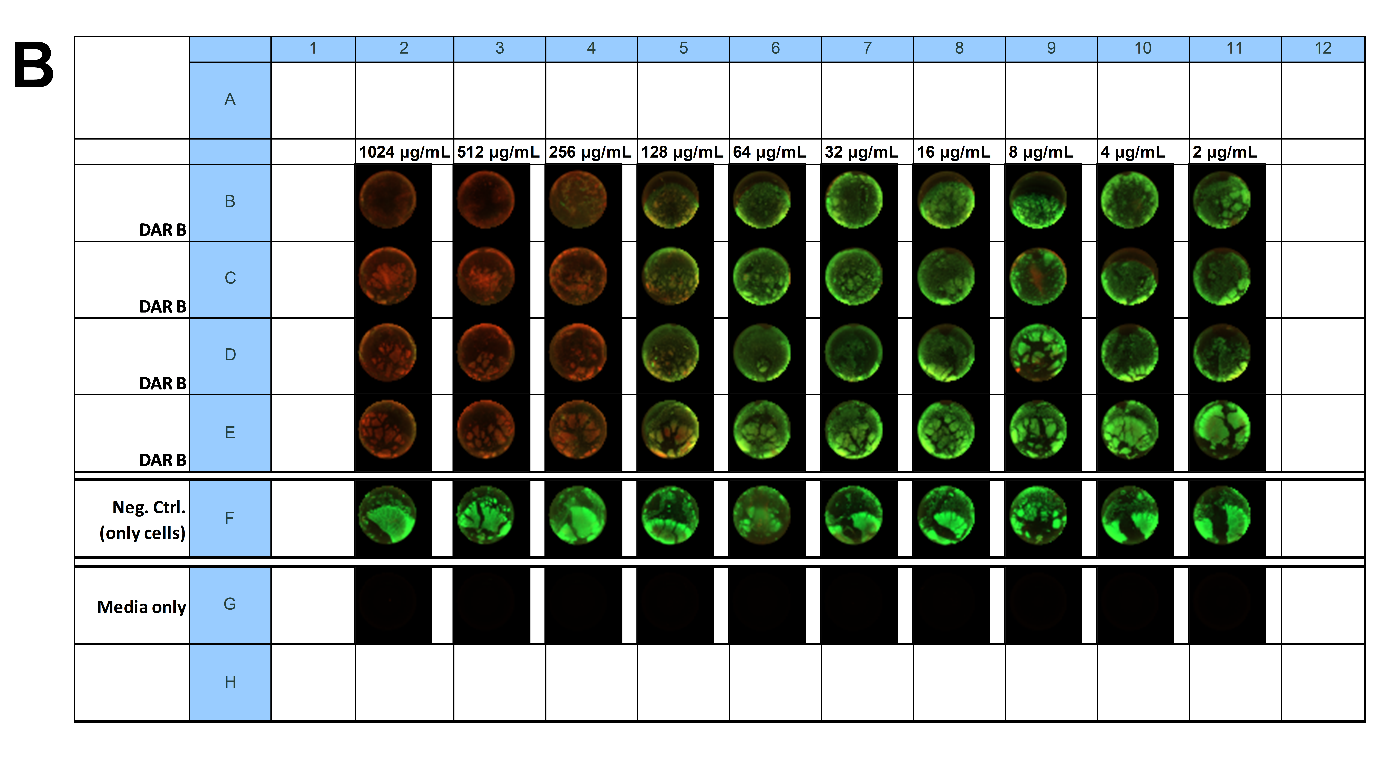


Figure S2 – Fluorescence images of the full assay plates of P. aeruginosa ATCC 27853 biofilms after 6-h treatment with colistin (A) and darobactin B (B). Antibiotics were tested in quadruplicate: Colistin was tested at 256–0.5 µg/mL in two-fold dilutions, while darobactin B was tested at 1024–2 µg/mL. Each plate assay contains 10 wells of untreated biofilms (as negative control) and media control.


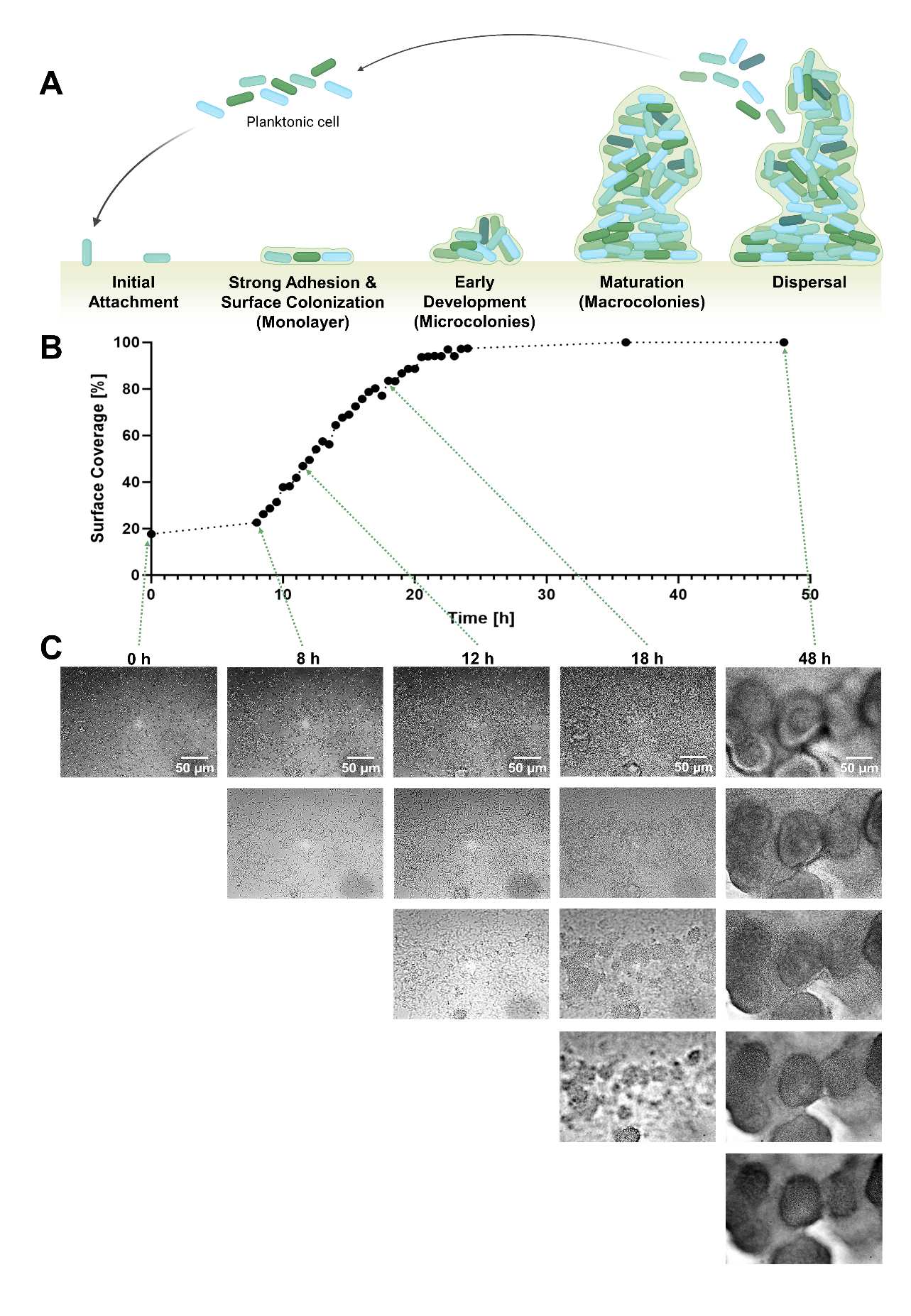


Figure S3 – Analysis of P. aeruginosa biofilm formation in a single location of the chip. A) Biofilm life cycle. Figure was created using BioRender^1^. B) Surface coverage of P. aeruginosa ATCC 27853 during the biofilm formation step. C) Microscopic images of various developmental stages of P. aeruginosa ATCC 27853 biofilms captured with different focal position to capture the 3D structure of the biofilms: initial attachment (0 h), strong adhesion (8 h), early developmental stage (microcolony formation, 12 h), maturation (macrocolony formation) (18 h and 48 h).


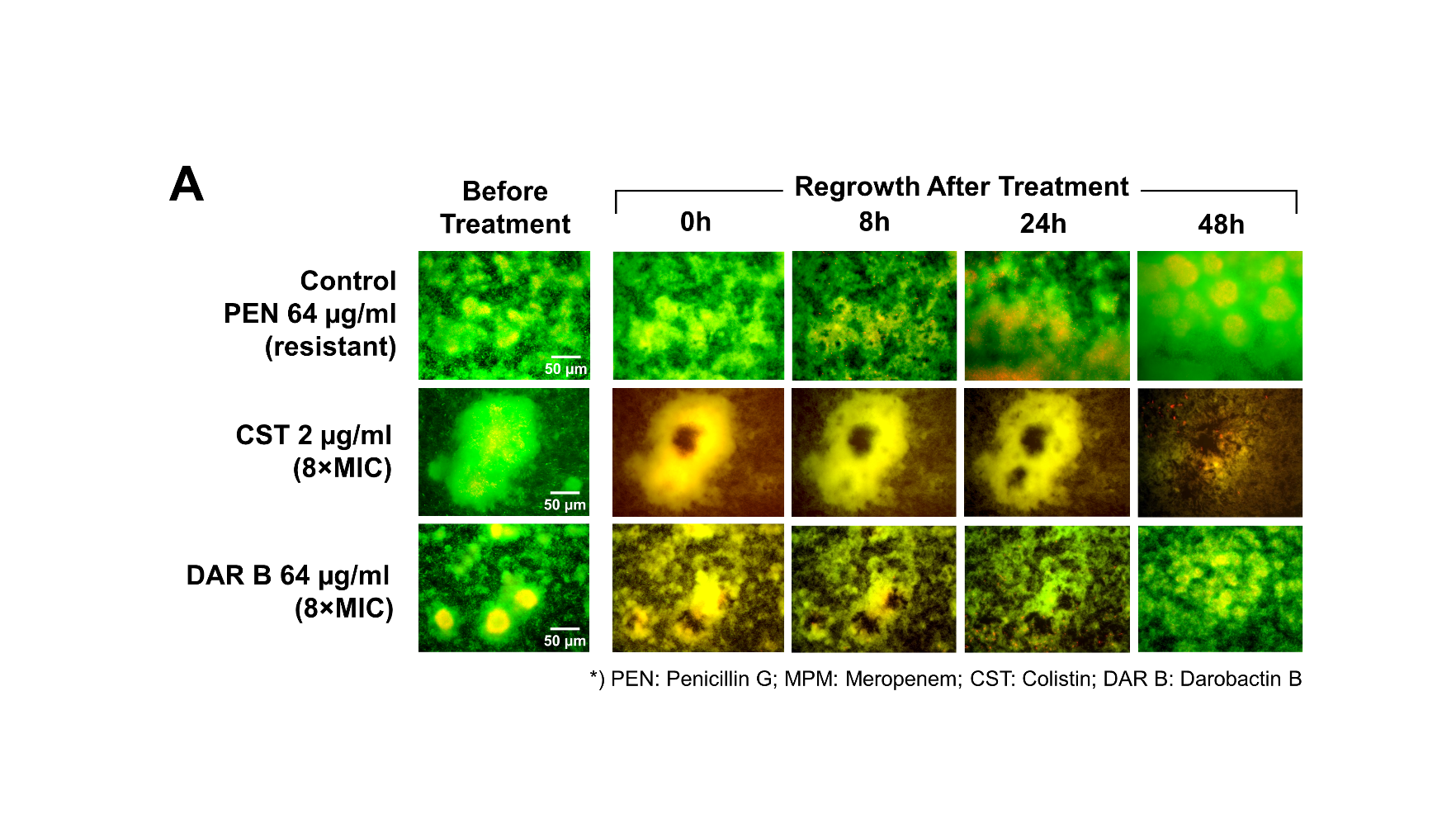


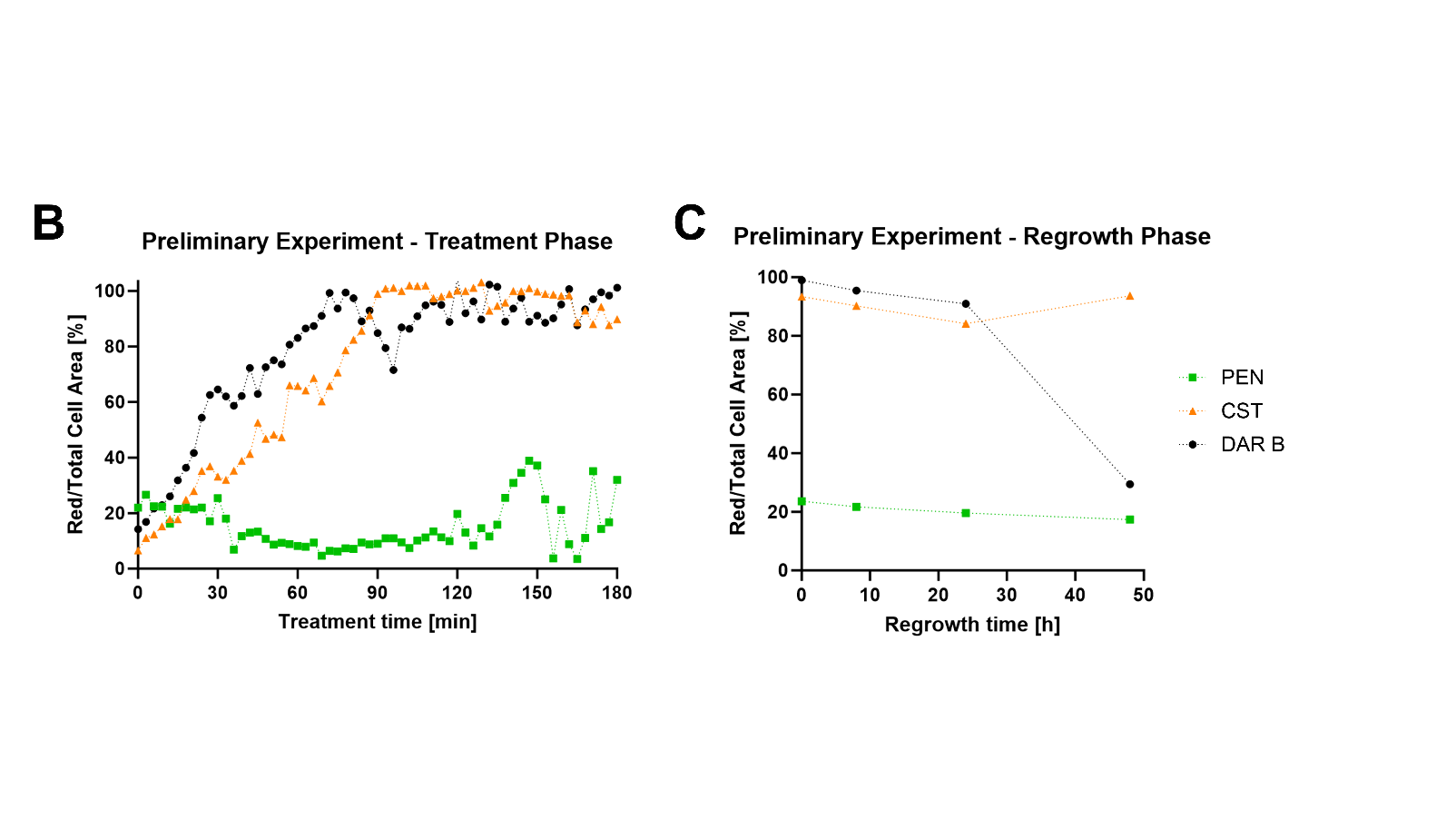


Figure S4 *–* (A) Observation of *P. aeruginosa* ATCC 27853 biofilms stained with SYBR Green I and PI before antibiotic treatment and during the regrowth phase, captured at one single location in the chip. Bottom: Effect of PEN, CST, and DAR B on mature *P. aeruginosa* ATCC 27853 biofilms during the 3-h treatment (B) and during the regrowth phase (C). Ratio of membrane-compromised (red-stained) cells to total cells was calculated over time during the treatment and regrowth phases.


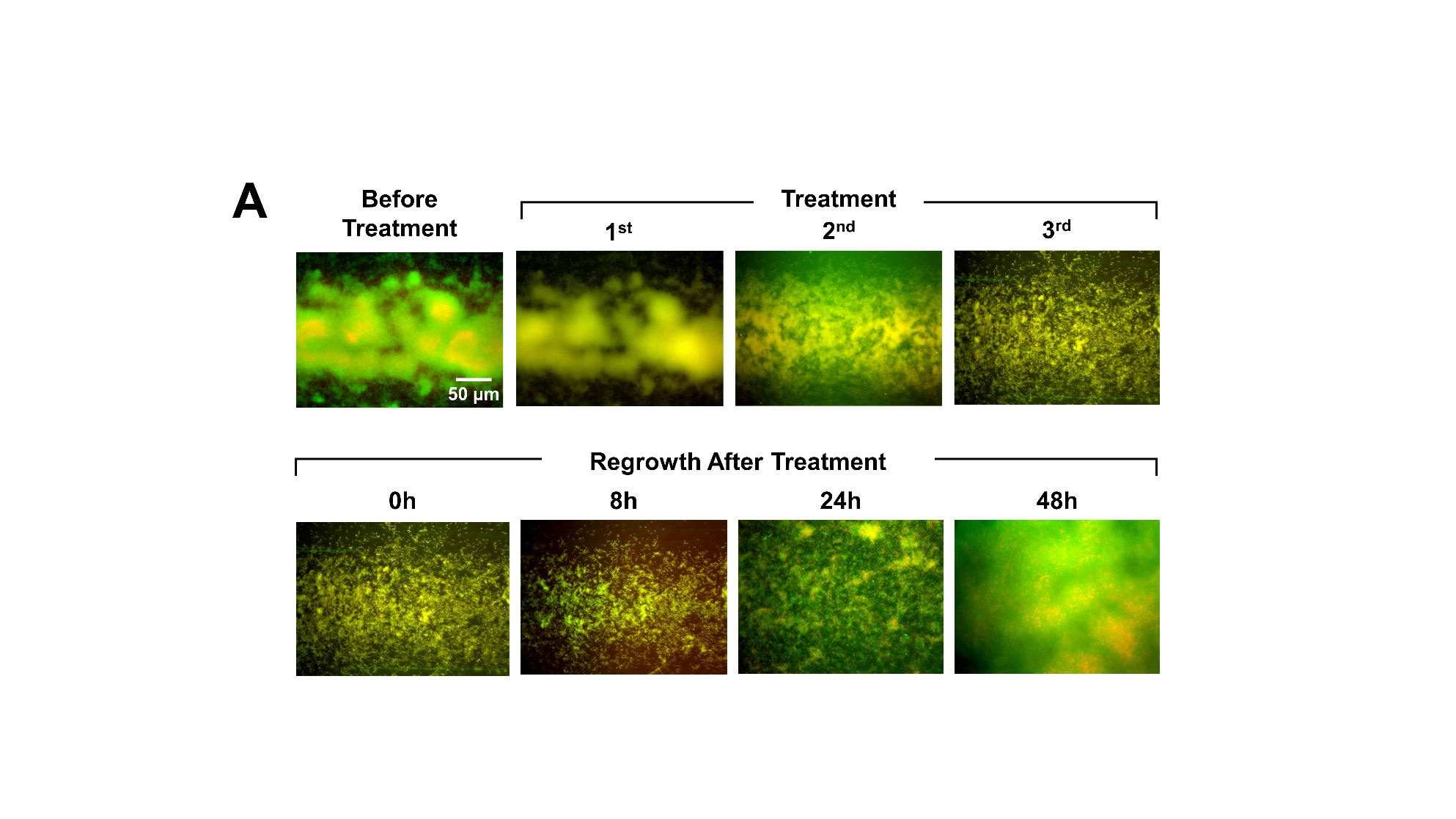


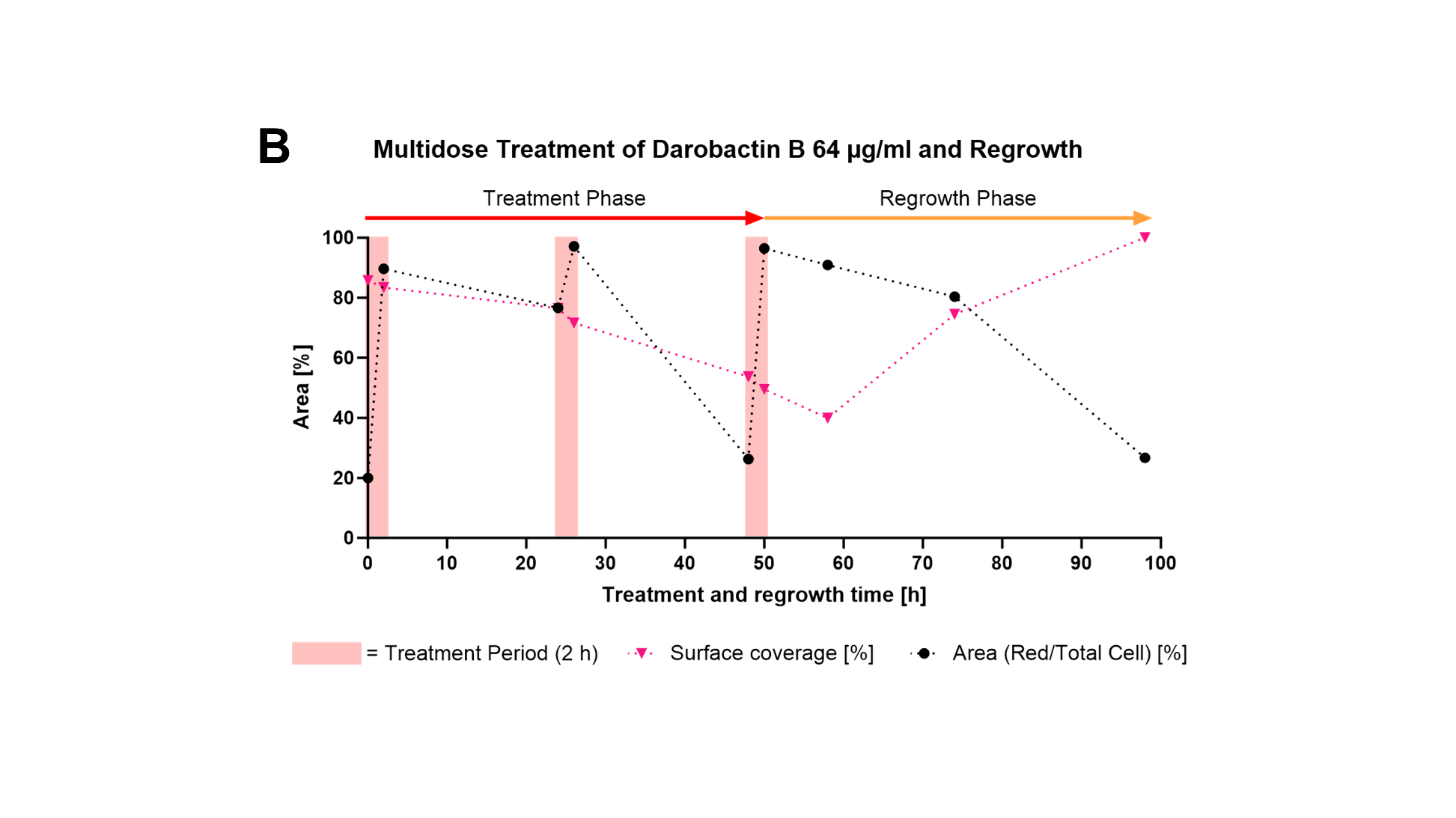


Figure S5 – (A) Observation of biofilms of *P. aeruginosa* ATCC 27853 stained with SYBR Green I and PI before treatment, after treatment with three doses of 64 µg/ml DARB, and during the regrowth phase. Each treatment involved the application of 64 µg/ml DARB for 2 h with 24-h interval. Fluorescence images were captured at one single location in the chip. (B) Effect of DAR B on mature *P. aeruginosa* ATCC 27853 biofilms during the sequential 2-h treatments and the regrowth phase, including the calculation of surface coverage and the ratio of membrane-compromised (red-stained) cells to total cells over time.


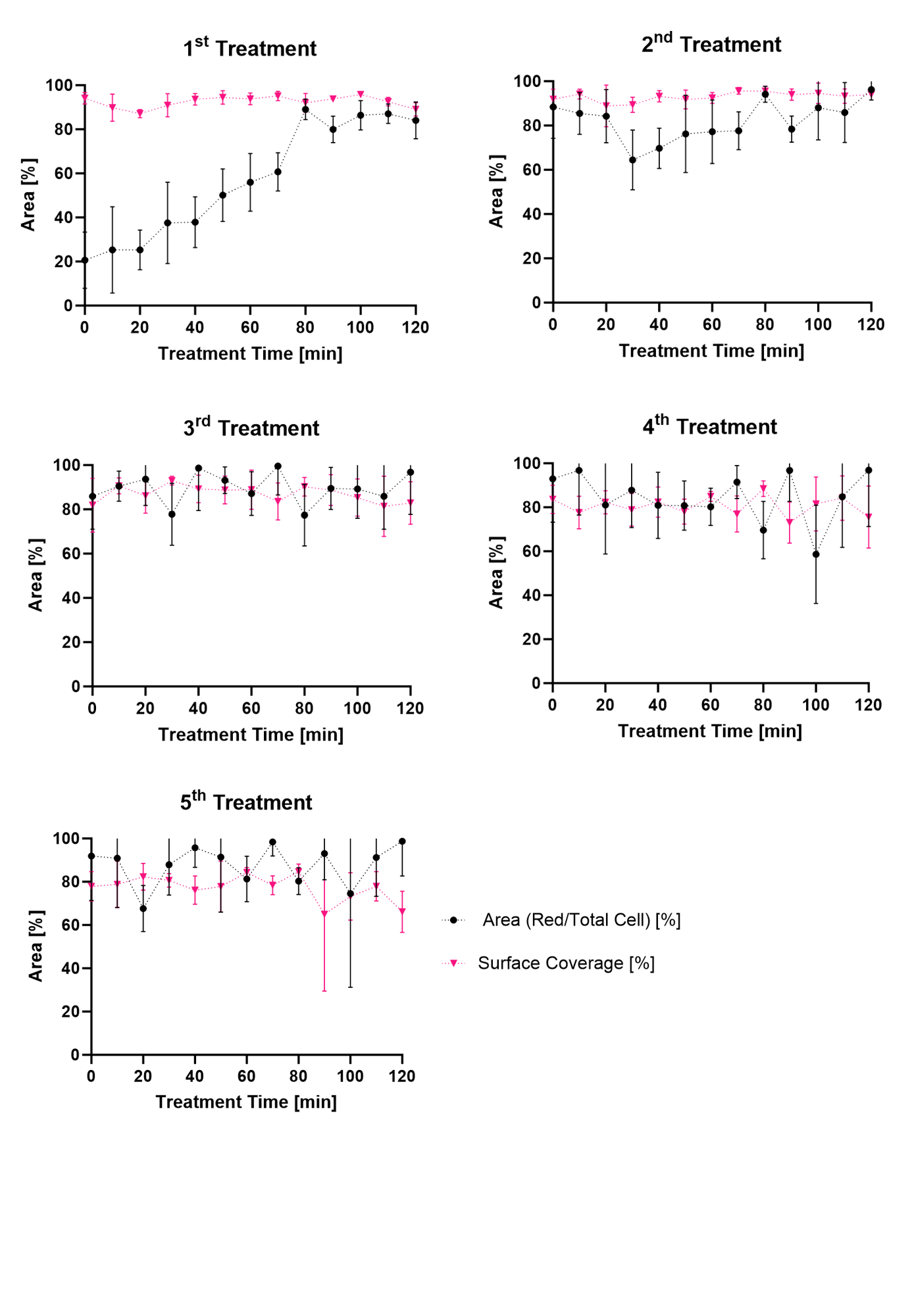


Figure S6 – Effect of multiple dosages of DAR B against the biofilms of P. aeruginosa ATCC 27853. Ratio of membrane-compromised (red) fluorescence signals and total cells as well as percentage of surface coverage over time were calculated for each 2-h treatment.

**REFERENCES**

1. Created in BioRender. Schäberle, T. (2025) https://BioRender.com/7kr7sff.
